# Interstitial lung disease in Systemic sclerosis: insights into pathogenesis and evolving therapies

**DOI:** 10.31138/mjr.29.3.140

**Published:** 2018-09-27

**Authors:** Sakir Ahmed, Sarit Sekhar Pattanaik, Mohit Kumar Rai, Alok Nath, Vikas Agarwal

**Affiliations:** 1 Department of Clinical Immunology, Sanjay Gandhi Postgraduate Institute of Medical Sciences, Lucknow, India; 2 Department of Pulmonary Medicine, Sanjay Gandhi Postgraduate Institute of Medical Sciences, Lucknow, India

**Keywords:** Interstitial lung disease, systemic sclerosis, progression

## Abstract

Interstitial lung disease (ILD) is a leading cause of mortality in systemic sclerosis (SSc). However, mortality is improving as pathogenesis is being better understood and new therapies emerge. The roles of the inflammasome and NETosis in fibrosis are being elucidated. Epigenetic targets like DNA methylation and microRNA show promise as new targets for anti-fibrotic agents. The IL17-23 pathway has been shown to be active in SSc-ILD. Newer biomarkers are being described like CCL18 and the anti-eIF2B antibody. Hypothesis-free approaches are identifying newer genes like the ALOX5AP and XRCC4 genes. Computer-aided interpretations of CT scans, screening with ultrasonography and magnetic resonance imaging (MRI) are gradually emerging into practice. Imaging can also predict prognosis. A plethora of studies has shown the benefit of immunosuppression in halting ILD progression. Extent of lung involvement and PFT parameters are used to initiate therapy. The best evidence is for cyclophosphamide and mycophenolate. Besides these, corticosteroids and rituximab are being used in cases refractory to the first line drugs. Stem cell transplant is also backed by evidence in SSc. Longer studies on maintenance therapy are awaited. The inflammation in SSc is mostly subclinical and there is great interest in developing anti-fibrotic drugs for SSc-ILD. Perfinidone and nintedanib are under trial. The last resort is lung transplantation.

## INTRODUCTION

The scenario for outcomes of SSc is no longer as bleak as it once was. Nihtyanova SI et al. have shown that survival has increased with better diagnosis and monitoring.^1^ The lung is the source of despair as well as hope in systemic sclerosis: despair, because most mortality can be attributable to the lung causes;^2^ and hope, because therapies that halt disease progress in SSc have mostly been described for interstitial lung disease (ILD). Clinically significant ILD has increased over the last two decades; in spite of this, the associated mortality has come down.^1^ ILD patterns on computerized tomography scans (CT) include certain patterns named as nonspecific interstitial pneumonia (NSIP), usual interstitial pneumonia (UIP), desquamative interstitial pneumonia (DIP), cryptogenic organizing pneumonia, diffuse alveolar damage, acute interstitial pneumonia, and lymphocytic interstitial pneumonia. The most common pattern in SSc is NSIP that has a prominent inflammatory component. The predictors for SSc-ILD are male gender and presence of anti-Scl70 antibodies while presence of anti-centromere antibodies is a protective factor.

In the present review, we searched Medline database for “Systemic sclerosis [MeSH] AND (interstitial lung disease OR ILD)” and manually screened for relevant articles looking into the pathogenesis, biomarkers, outcome and therapies of SSc-ILD, utilising a previously published search strategy for narrative reviews.^3^

## PATHOGENESIS

SSc may be the phenotypic expression of the result of various different aetiopathological processes. Possible evidence for such a hypothesis is the Erasmus syndrome. Reported in less than 1% of SSc patients, it is described as the development of SSc after exposure to silica.^4^ But, in majority of SSc, no clear aetiological factor can be found.

**Figure 1.**
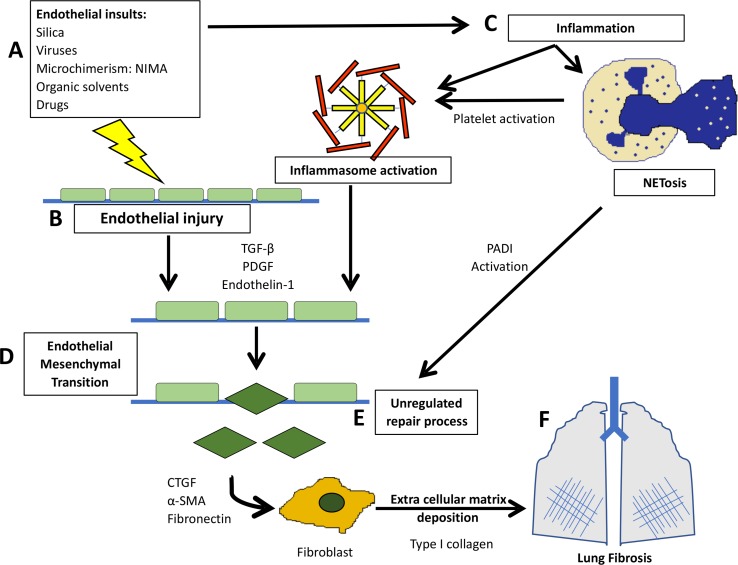
In a background of genetic susceptibility, various environmental insults (A) lead to (B) endothelial injury and (C) Inflammation. (B) Endothelial activation leads to conversion of endothelial cells into mesenchymal progenitors (D). Also (C) inflammation causes NETosis and inflammasome activation that initiate a repair process that is imprinted on the fibroblasts (E). All these lead to conversion of fibroblasts into myofibroblasts and extracellular matrix deposition in the lung interstitium (F) and thus fibrotic lung disease. (NIMA: Non-inherited maternal antigens; TGF-β: Transforming growth factor-β; PDGF: Platelet derived growth factor; CTGF: Connective Tissue Growth Factor; α-SMA : α- Smooth muscle actin; PADI: Peptidyl arginine deiminase)

The trigger for onset of fibrosis may be an injury either to the lung epithelium or to the endothelium of the supplying vessels. In SSc there seems to be an altered and unregulated repair process. As in the skin and other organs, the lung also has unregulated repair leading to fibrosis. This leads to overproduction of signalling peptides like transforming growth factor-β (TGF-β), endothelin-1, platelet derived growth factor (PDGF), etc. TGF-β has both canonical and non-canonical downstream pathways and both have been implicated in fibrosis. Under the influence of these cytokines, endothelial cells are supposed to have a mesenchymal transition (EMT). Ultimately, genes like type I collagen, fibronectin, α-smooth muscle actin and connective tissue growth factor (CTGF) are over-expressed leading to extracellular matrix deposition (**Figure 1**).

Newer concepts in fibrosis include the role of the Inflammasome and of NETosis. Inflammasomes activate pro-IL1β to IL-1β initiating an inflammatory cascade. Silencing of NLRP3 (component of the inflammasome) in a bleomycin model of fibrosis reduced levels of TGF-β, E-cathedrin and α-smooth muscle actin; thus, it may influence epithelial-mesenchymal transition that is the forerunner of fibrosis.^5^ NETosis has been reported to increase organ fibrosis in aging mice.^6^

Shifting from genetics to epigenetics has revealed the importance of microRNA like miR-155. miR-155 has been reported to influence NLRP3 inflammasome activation.^7^ The other facet of epigenetics is DNA methylation. It has been shown that hypomethylation of integrin genes^8^ occurs in SSc that may lead to increased TGF-β expression^9^ and myofibroblast differentiation.^10^ These have opened up new avenues for targeting fibrosis in SSc.

Interestingly, a study on IL17-23 pathway observed that IL-17 and IL-23 were reduced and IL-21 was elevated in SSc as compared to sera of healthy controls.^11^ However, in exhaled breath of SSc-ILD patients, there was increased TNF-α, TGF-β, IL-17 and IL-23; possibly implying a possible local role of this axis in the pathogenesis of fibrosis.^12^

## BIOMARKERS

The clinical biomarker for SSc-ILD has been the six-minute walk test, originally described for use in patients with pulmonary hypertension. A meta-analysis on the six-minute walk test showed it to be a validated marker for SSc-ILD with or without pulmonary arterial hypertension (PAH).^13^

Autoantibody production is a predictor of internal organ involvement in SSc. Classically; the strongest risk has been described with anti-topoisomerase (anti-Scl-70) antibodies, whereas anti-centromere antibodies are likely protective. A serum immunoprecipitation study of 548 SSc patients has revealed a new antibody directed against Anti-Eukaryotic Initiation Factor 2B (Anti-eIF2B) in seven patients. There was correlation of this antibody with presence of ILD and diffuse disease.^14^

Previously established serum biomarkers include surfactant proteins A and D and Krebs von den lungen-6 (KL-6). KL-6 is a glycoprotein that is expressed by type II pneumocytes, and especially found in areas of tissue injury in the lung. It can help in screening for ILD, and has good utility in evaluating disease activity and predicting prognosis in SSc-ILD.^15^ CCL18, previously known as macrophage inflammatory protein-4 (MIP-4), attracts naïve B lymphocytes and immature monocyte-derived dendritic cells to germinal centres. Normal lung does not have CCL18 gene transcripts.^16^ Thus, its presence may imply ongoing inflammation and perpetuation of fibrosis. Recently, high serum CCL18 was shown to predict highest rates of annual deterioration of forced vital capacity (FVC). It was reported that ILD patients with high CCL18 also had lower 5-year and 10-year survival compared with patients with low CCl18 levels.^17^

The rs10507391 polymorphism (T/A) of arachidonate 5-lipoxygenase activating protein (ALOX5AP) was identified in the EUSTAR cohort to predict the risk of SSc (odds ratio [OR] 1.27 [95% CI 1.07, 1.50], P < 0.05 vs controls) as well as that of ILD in SSc (OR 1.45 [95% CI 1.17, 1.79], P < 0.05 vs SSc patients without interstitial lung disease).^18^ A whole exome sequencing of 32 patients found BANK1 and TERT to be over-expressed. These had already been described in ILD and in SSc. However, the new discovery was over-expression of the 2-LTR circle formation pathway, mainly the XRCC4 DNA repair gene.^19^

## IMAGING

In the field of imaging, computer-aided analysis of computerized tomography (CT) scan of ILD and use of ultrasound (USG) for lung imaging are emerging. Conventional visual reader-based score (CoVR) with a computer-aided diagnosis (CaM) were comparable in a study and correlated with pulmonary function test (PFT) parameters, Borg dyspnea score and Health assessment questionnaire disability index (HAQ-DI).^20^ Even difference with treatment can be quantified by computer-aided analysis.^21^

PFT is much less sensitive than CT in picking up ILD,^22^ but the question remains if the exposure to radiation is justifiable by the benefits of detecting asymptomatic ILD. MRI technology is catching up with CT for ILD. Though still inferior to CT, current high-Tesla MRI machines can offer a radiation-free alternative for ILD detection.^23^

A promising screening strategy for ILD can be the use of ultrasound (US). The number of B-lines (“comet” artifact) has shown correlation with CT scores^24^ in SSc. A review has looked into different scoring systems for lung USG, but points out that limitations remain. Firstly, the origin of B-lines are still debated, and secondly, comprehensiveness of a scan correlates inversely to the time spent on the scan.^25^ Other factors predicting ILD on US include blurring or irregularities of the pleural line and subpleural consolidations.^26^

## OUTCOMES AND PREDICTORS

As we have seen, serum markers like CCL18 and non-irradiating imaging like MRI and USG hold a promise for screening for SSc-ILD. They can also predict mortality and morbidity. However, this is based on preliminary data only, and need to undergo further evaluation before coming to clinical practice.

Acute exacerbations of SSc-ILD are not uncommon and presence of dermatomyositis or polymyositis overlap predicts higher risk of acute exacerbations.^27^ Short term changes in PFT can predict long-term outcome including mortality: a fall of Forced vital capacity (FVC) >10% or a fall of DL_CO_ >15% with a fall in FVC of 5–9% seemed to be the best predictor in a study.^28^

Besides presence of ILD, predictors of mortality in SSc include male gender, age more than 65 years at onset, presence of digital ulcers, pulmonary hypertension, cardiac involvement, scleroderma renal crisis (SRC), presence of anti-topoisomerase I and absence of anti-centromere antibodies, and an active pattern on capillaroscopy.^29^

Analysis of the placebo arm of the SLS1 study has shown that ILD progresses independent of the disease duration and the best predictor of rate of progression is the quantity of fibrosis on CT underscoring the need for treatment.^30^

## THERAPY

At present, no treatment has shown to reverse lung fibrosis in a patient with SSc-ILD. Thus, it is of paramount importance to understand the risk of progression in order to decide when therapy should be started. Since immunosuppressive therapies represent a double-edged sword with increased incidence of infections in SSc-ILD, their use should be rationalised. It may not be ethical to treat an early ILD who has no progression, but no deserving patient should be denied therapy. Thus, in real life there is always a dilemma, as we have no definite marker to predict ILD progression.

Most authors support treatment according to the following criteria:^31^ (i) all patients presenting at first observation with either an extent of lung disease >20% on HRCT or an indeterminate extent of disease plus an FVC < 70%, and (ii) during follow-up, all patients experiencing a significant decrease of DLCO (>15%) or FVC (>10%) or both, whatever the extent of lung involvement. In both, the SLS studies only symptomatic patients (at least grade 2 on Mahler Dyspnea Index) were recruited. In the treatment of ILD in SSc commonly used drugs are corticosteroids, cyclophosphamide, mycophenolate, azathioprine, and rituximab. The evidence for each is examined below. Most of these studies deal with induction therapy. Maintenance studies with longer follow-up are required.

Trials with biological therapies (abatacept, tocilizumab and anti-tumour necrosis factor [(TNF)-α]), etc. are underway. However, they are for SSc and not specifically for SSc-ILD. These therapies are not recommended outside a clinical trial.

A small study classifying SSc-ILD into usual intersitital pattern (UIP) versus non-UIP found that UIP pattern had a poorer outcome with immunosuppression.^32^

### 1. Cyclophosphamide

It was the first evidence-based therapy for SSc-ILD. Two double-blind^33,34^ and one unblinded^35^ randomised controlled trials (RCTs), have shown a significant increase in FVC in patients treated with cyclophosphamide (CYC). (**Table 1**)

**Table 1.** Randomised controlled trials in patients.

**Drug**	**Year of publication**	**Inclusion**	**Outcome**	**Remarks**
CYC plus AZA versus placebo	2006, RCT ^34^	45 SSc-ILD	No difference in outcome though trend towards significance in CYC group	38% lost to follow-up
Oral CYC versus AZA	2004, Unblinded RCT ^35^	60 SSc (30 each arm)	No change in FVC or DL_CO_ in CYC group Deterioration in AZA group	Number of patients with ILD not mentioned
CYC versus placebo	2007, RCT ^36^	158 SSc-ILD (evaluated 72 CYC; 73 placebo)	Beneficial effects on PFT persisted only till 18 months though subjective effects on dyspnea perisisted for 2 years	Second year follow-up of SLS1 study
MMF vs CYC	2016, RCT ^38^	63 MMF; 63 CYC	No statistical difference in adjusted FVC between groups except greater number of withdrawals and failed treatment with CYC	Both MMF and CYC improved lung function, imaging, dyspnoea and skin disease

Scleroderma lung study (SLS I) was the first RCT to demonstrate efficacy of oral CYC. Patients had a modest improvement in FVC as compared to placebo which persisted for 18 months after discontinuing treatment, but effect was lost by 24 months. This reiterates the requirement for some maintenance therapy. Apart from the primary endpoint, there was significant improvement in a number of secondary endpoints like total lung capacity (TLC), the patient-reported outcome of dyspnea (Mahler Dyspnea Index), and several quality of life measures. Furthermore, a retrospective, multivariate regression analysis of SLS I found patients with maximal severity of reticular infiltrates on chest HRCT, high mRSS scores and the Mahler baseline dyspnea index at baseline had better response to CYC compared to those with less severe fibrosis on HRCT.^36^

In the Fibrosing Alveolitis in Scleroderma Trial (FAST) trial, patients with SSc-ILD were randomized to receive either intravenous cyclophosphamide (600 mg/m^2^ monthly) for 6 months followed by daily oral azathioprine 2.5 mg/kg/d (maximum 200 mg/d), or placebo infusions followed by oral placebo.^34^ At 12 months, a modest but statistically non-significant improvement in FVC was seen in the actively treated group.

Toxicity is an important factor in patients treated with CYC: in the short-term, this includes leukopenia and infections, while long-term effects include infertility, bone marrow toxicity and carcinogenesis. However, in light of evidence in favour of CYC, the EULAR Scleroderma Trials and Research (EUSTAR) group recommends CYC for the treatment of SSc-ILD.^37^

### 2. Mycophenolate Mofetil

Earlier observational studies had shown the effectiveness of Mycophenolate in treatment of SSc-ILD. The SLS II study, a randomised, double-blind, parallel group trial comparing mycophenolate at dose of 1.5gm twice daily for 24 months with oral CYC for 12 months resulted in significant improvements in prespecified measures of lung function over the 2 year course of the study in both the arms. Although mycophenolate mofetil was better tolerated and associated with less toxicity, the hypothesis that it would have greater efficacy at 24 months than CYC was not confirmed. These findings support the potential clinical effectiveness of both CYC and mycophenolate mofetil for progressive SSC-ILD. The present preference for mycophenolate mofetil is possibly because of its better tolerability and toxicity profile.^38^ An interesting observation was that deaths with CYC was 10/73 versus 5/69 with MMF. Though not statistically significant, the SLSII study was not powered to look at a difference in mortality.

### 3. Azathioprine

In an RCT comparing azathioprine with CYC as first line of therapy for SSc-ILD, a decline in FVC and DLCO at 10 months was observed in the azathioprine group.^35^ However, studies are encouraging with regard to its use in maintenance phase particularly following CYC induction.^39,40^

### 4. Corticosteroids

Corticosteroid pulses have been used in association with CYC in the treatment of ILD-SSc with favourable results. However, a word of caution is advised while using corticosteroids in SSc patients in view of precipitating scleroderma renal crisis. A small study comparing CYC (1g/m^2^) versus CYC with high dose prednisolone found no benefit of adding prednisolone.^41^

### 5. Rituximab

A pilot study of 14 patients with diffuse cutaneous SSc given two cycles of rituximab (at baseline and 24 weeks) showed significant improvement in FVC & DLCO at 2 years.^42^ The EUSTAR group evaluated rituximab in a nested case-control designed study and observed a stabilization of FVC and improvement in DLCO in SSc-ILD patients on rituximab compared to placebo.^43^ This is an upcoming option for SSc-ILD and needs further validation by RCTs like the ongoing RECITAL trial before recommendations can be made for routine use.^44^

### 6. Autologous Hematopoietic Stem Cell Transplantation

Intense immunosuppression followed by hematopoietic stem cell transplantation (HSCT) has emerged as a new therapeutic modality in treatment of SSc-ILD. First ASSIST (Autologous Stem Cell Systemic Sclerosis Immune Suppression Trial), a single-centre, open-label phase II trial of autologous HSCT *without* CD34+ cell selection, had showed improvement in comparison to CYC.^45^ The ASTIS (Autologous Stem cell Transplantation International Scleroderma) and SCOT (Scleroderma: Cyclophosphamide Or Transplantation) trials have compared the safety and efficacy of HSCT versus monthly pulse intravenous CYC. In ASTIS, patients treated with autologous HSCT experienced more deaths and other adverse events in the first year, but had improved long-term event-free survival compared with patients treated with CYC.^46^ The SCOT trial has shown unprecedented success with superior event free survival (74% with SCT versus 47% with CYC) and overall survival (86% with SCT versus 51% with CYC) at 72 months.^47^

Other trials on HSCT like STAT (Scleroderma Treatment With Autologous Transplant) trial are ongoing. Future studies on larger patient groups will determine if this modality becomes routine procedure in management of patients with SSc-ILD. However, the success of less toxic therapies may also relegate it to the background.

### 7. Anti-Fibrotic Therapy

Given the role of fibrosis in SSc ILD, anti-fibrotic agents are definitely on the cards for future drug development. Perfinidone and nintedanib, both FDA approved for idiopathic pulmonary fibrosis (IPF), are now undergoing studies in SSc-ILD patients. The SENCIS trial is a phase III trial of nintedanib in SSc-ILD that is currently ongoing.^48^ Many other new therapeutics that target specific growth factors, cytokines or pathways (e.g., monoclonal CTGF antibodies, tocilizumab, endostatin 1-derived peptide, caveolin scaffolding domain), as well as multiple existing drugs that might be repurposed to treat fibrosis (e.g., PPAR-γ agonists [e.g., rosiglitazone], statins [rosuvastatin], fluoroquinolone antibiotics [e.g., ciprofloxacin], and thrombin inhibitors [e.g., dabigatran]) are being tried for SSc-ILD.

In a study of imatinib in 30 patients unresponsive to CYC, 50% were shown to have stable disease while four had improvement in FVC (>15%). However there was no placebo arm and the late effect of CYC cannot be ruled out.^49^ Even combination of imatinib with CYC has been tried in a small cohort but failed to show much benefit.^50^ Bosentan had been tried for anti-fibrotic effects in ILD but it did not have benefit.^51^

### 8. Lung Transplantation

Lung transplantation is a life-saving option for SSc-ILD patients who are not responsive to medical treatment. Unfortunately, lung transplantation is not always possible due to the involvement of other organs. The survival is similar in patients of IPF, but recently specific SSc contraindications have been proposed. They include active inflammatory myopathy, active digital ulcers, severe gastrointestinal involvement, cardiac arrhythmias, unstable renal function in the past 3 months, an interval of <3 years between scleroderma renal crisis (SRC) and transplantation.^52^

## CONCLUSION

Thus, the landscape for SSc-ILD is evolving within newer insights into pathogenesis, newer and better biomarkers, and the ingress of USG and MRI to reduce exposure to ionizing radiation. There is a plethora of immunosuppressive drugs that are being tried for SSc-ILD with varying degrees of success. But success until now is defined in arresting the disease. Fibrosis still cannot be reversed. With these newer insights into the pathogenesis, possibly we can look forward to novel approaches to SSc-ILD therapy in the coming decade.
